# Parents’ Perspectives on Early Childhood Oral Health Care—Results from a Survey in a Vulnerable Population in Palos de la Frontera (Huelva, Spain)

**DOI:** 10.3390/healthcare14111442

**Published:** 2026-05-23

**Authors:** María Rosado Moreno, Leydi Bech Barcaz, Asunción Mendoza Mendoza, Antonio Castaño Seiquer, David Ribas-Pérez

**Affiliations:** 1 Departamento de Estomatología, Universidad de Sevilla, 41009 Seville, Spain; mrmariarsd@gmail.com (M.R.M.); amendoza@us.es (A.M.M.); acastano@us.es (A.C.S.); 2 Andalusian Health Service, 41071 Seville, Spain; bechbarcaz@gmail.com

**Keywords:** child, oral health, socioeconomic factors, childhood caries

## Abstract

Introduction: Oral health in early childhood is essential. Parents, as the primary caregivers, must possess basic knowledge to achieve optimal oral health status. Objectives: The study aims to assess parents’ knowledge and perspectives on early childhood oral health and to evaluate how nationality and educational attainment influence their preventive habits and dental priorities within a nursery and primary school in the province of Huelva (Spain). Materials and Methods: The sample consisted of 129 parents of children aged 3, 4, and 5 years from the aforementioned educational center. A modified questionnaire, validated by experts in the field, was used as the assessment tool. Results: Nationality and education were key determinants of oral health literacy. Spanish-born guardians reported significantly higher dental attendance for their children compared to foreign-born guardians (97.7% vs. 84.2%; *p* = 0.030). A profound cultural gap was observed in caries etiology: 71.1% of foreign-born respondents attributed caries to “infections or heredity,” while 98.4% of Spanish-born respondents correctly identified behavioral factors (*p* < 0.001). Regarding educational attainment, 75% of the high-education group prioritized functional health (posterior sector) compared to only 26.3% in the low-education group (*p* < 0.001). Additionally, a non-linear gap was found in knowledge of primary tooth complications, with the medium-education group showing the lowest awareness (34.8%; *p* = 0.047).

## 1. Introduction

Oral health in childhood is fundamental for a child’s integral development; however, pathologies such as Early Childhood Caries (ECC) remain prevalent [1,2]. Parents, as those primarily responsible for childcare, play a crucial role in preventing these pathologies through hygiene habits and early access to dental services. Nevertheless, lack of information, limited knowledge, or sociocultural barriers often hinder clinical practice [3,4,5].

Frencken et al. (2017) performed a review of global prevalence and incidence of dental caries [6]. It revealed that over the past 40 years, dental health has improved in some ways—especially among children. Fewer 5- and 12-year-olds have severe tooth decay, and people are keeping more of their natural teeth worldwide. High-income countries show the lowest levels of tooth decay, particularly among 12-year-olds and adults aged 35–44. But, at the same time it still remains one of the most prevalent diseases globally, especially in some vulnerable groups [2,6].

The magnitude of this issue needs significant resource investment in treatments that could be avoided by increasing preventive measures [7]. Health professionals must inform and encourage parents or legal guardians at an early stage regarding oral health care as an integral part of their children’s overall health. Routine dental check-ups are essential [8]. Current clinical guidelines emphasize that parents should implement specific evidence-based preventive measures to ensure early childhood oral health. These include establishing oral hygiene routines as soon as the first tooth erupts, ensuring a first dental consultation within the first year of life for professional guidance, and managing dietary habits by reducing fermentable carbohydrate intake [9,10]. Furthermore, avoiding horizontal transmission of cariogenic bacteria through cross-contamination (e.g., sharing utensils or cleaning pacifiers with saliva) remains a critical preventive strategy [9]. The extent to which parents understand and adhere to these specific practices is a key indicator of oral health literacy and forms the basis of the assessment in this study.

In the Western world, as in many other societies, dentistry is predominantly practiced in private clinics and, to a lesser extent, in the public sector. This dynamic often reproduces social inequalities, as access depends on the family’s economic capacity. Factors such as socioeconomic status, culture, ethnicity, gender, religion, language, and disability must be considered to ensure equity in oral health [11,12].

Regarding the context in Spain, dental practice was almost exclusively private until 1980. Significant efforts to reduce social disparities were made through the implementation of community pediatric programs, such as the Dental Care Program for Children (PADI—Programa de Atención Dental Infantil). While Spain has achieved low or very low caries indices, this does not apply uniformly across the population. Marginalized groups continue to accumulate a high percentage of oral pathologies due to cultural factors, language barriers, or limited resources [10,13]. Significant efforts to reduce social disparities were made through the implementation of the PADI program. In Spain, while the National Health System provides universal coverage, dental care for adults is largely private. To address this, the PADI was established as a regional-level initiative—pioneered in the Basque Country and Navarra, and subsequently implemented in Andalusia in 2002—to provide free basic dental care for children aged 6 to 15. The program is managed by the Regional Ministry of Health (Consejería de Salud de la Junta de Andalucía) and operates through a mixed-management model involving both public health centers and accredited private dental clinics [10,13,14]. This framework aims to ensure equity in access, regardless of the family’s socioeconomic status, although its focus on permanent dentition often leaves a preventive gap in early childhood (ages 0–5), which is the focus of the present study.

Caries remains the most common oral disease in the country. In the 5–6-year age group, it significantly affects those from lower socioeconomic backgrounds (38.3% vs. 15.6% in high levels) and those born outside Spain [13].

This situation is specifically reflected in Andalusia, where the latest Epidemiological Study of School Oral Health in Andalusia reflects that caries distribution is not uniform and it shows a strong association with socioeconomic status. Those in disadvantaged situations present nearly double the prevalence and dft/DMFT indices compared to their high-income counterparts. Furthermore, urban residents show lower prevalence and caries indices than those in rural or intermediate density areas. The latest Epidemiological Study of School Oral Health in Andalusia reported a caries prevalence in the primary dentition of 42.3% at age 6, a figure significantly higher than the national average. Furthermore, the Significant Caries Index (SiC) in this region reveals that a polarized 25% of the pediatric population concentrates more than 75% of the total disease burden. These figures underscore that, despite the PADI program’s efforts, early childhood caries remains a major public health challenge in the region, particularly in areas with high migrant populations and lower educational attainment, such as the province of Huelva [13,14].

A robust body of evidence demonstrates a direct correlation between parents’ oral health knowledge and practices and the prevalence of dental caries in their children [8,15]. Specifically, parental health literacy acts as a social determinant that shapes the early home environment, influencing dietary habits and the establishment of hygiene routines during the critical first years of life [15]. Effective supervision and the establishment of self-care habits during the first years of life are essential for improving long-term oral health [16].

The objective of this research is to evaluate the perspectives and knowledge of parents and guardians regarding oral health care in early childhood, specifically analyzing the influence of nationality and educational attainment on these perceptions. Furthermore, the study aims to determine whether parents prioritize aesthetic appearance over functional oral health in a vulnerable population in Andalusia (Spain).

To guide this investigation, the following hypotheses were formulated:

**H1.**  *There is a significant association between the guardian’s nationality and their knowledge of caries etiology, with foreign-born parents showing a more fatalistic or hereditary view of the disease.*

**H2.**  *Higher educational attainment in parents correlates with a greater prioritization of functional and posterior dental health over purely aesthetic concerns.*

**H3.**  *Socioeconomic and cultural barriers (such as nationality) significantly limit the frequency of preventive dental visits in the study population.*

## 2. Materials and Methods

### 2.1. Study Type and Settings

A descriptive, observational, cross-sectional study was conducted to assess oral health knowledge and practices among parents in a nursery and primary school in Palos de la Frontera (Huelva, Spain). This design was chosen as it allows for the analysis of the prevalence of knowledge and the association between socio-educational variables and preventive habits.

The Google Forms platform was used for data collection. Data were compiled in Excel and analyzed using SPSS software (IBM SPSS Statistics (version 27.0; IBM Corp., Armonk, NY, USA)) for efficient statistical processing. This cross sectional study has followed the STROBE (Strengthening the Reporting of Observational Studies in Epidemiology) guidelines for observational research [17].

### 2.2. Sample Selection

The final sample included 129 parents of children in the Early Childhood Education level (ages 3, 4, and 5). This age range was chosen because permanent teeth typically erupt at age 6.

Sampling Strategy: A non-probabilistic purposive sampling (convenience sampling) was employed. This strategy was selected due to the specific focus on a vulnerable population and the logistical constraints in accessing official census data from the educational center. To minimize selection bias, the survey was distributed to all parents across all classroom groups in the Early Childhood Education level through multiple official and informal communication channels (iPASEN and class delegates).

Sample Size and Power: The final sample size was *n* = 129 participants. Considering the total enrollment of the selected school for that age group (approximately 180–190 children), this sample represents over 65% of the target population. To ensure the statistical validity of the bivariate analysis, a post hoc power analysis was considered. For a medium effect size (Chi-square) and a significance level of α = 0.05, a sample of 129 is sufficient to achieve a statistical power (1-β) exceeding 80%, which is the standard threshold for identifying significant associations between variables such as nationality, education, and oral health knowledge.

### 2.3. Questionnaire

The instrument used was a questionnaire originally developed by the Pontifical Catholic University of Chile [18], which was subsequently adapted and validated for the Spanish context through a two-stage process. First, the content validity was assessed by a panel of three experts (two pediatric dentists and one public health specialist), who evaluated the relevance and clarity of the items, resulting in the modification of four questions to align with local terminology (e.g., ‘PADI’ and ‘guardería’). Second, a pilot study was conducted with a small sample (*n* = 10) of parents from a different school to ensure comprehensibility. The internal consistency of the knowledge-based questions was verified using Cronbach’s alpha, yielding a value of 0.78, which indicates acceptable reliability for this type of observational research.

It was anonymous, self-administered, and distributed electronically. The final questionnaire consisted of 18 items divided into 3 blocks, with an average completion time of 1.5 to 2 min.

The questionnaire was structured into three thematic blocks. Block A collected sociodemographic data, including the child’s age, the respondent’s nationality, and their highest level of educational attainment. Block B assessed knowledge regarding oral health maintenance, the etiology of dental caries, the importance of primary dentition, and the role of fluoride. Finally, Block C evaluated preventive habits and parental priorities, focusing on dental visit frequency, hygiene routines, and the perceived value of aesthetic versus functional dental sectors.

A pilot study was initially conducted among 10 legal guardians of patients at the Master’s Program in Pediatric Dentistry (Faculty of Dentistry, University of Seville). This phase aimed to ensure the clarity of each item and to identify and mitigate potential biases. Subsequently, formal authorization was obtained from the Director of a public nursery and primary school in Palos de la Frontera, Huelva. This collaboration was essential for establishing effective communication with the target population (legal guardians).

The survey link was distributed via mobile messaging applications (WhatsApp, v26.9.1; Meta Platforms, Inc., Menlo Park, CA, USA) through class delegates. Additionally, an official notification was issued via iPASEN, the communication and management platform of the Regional Ministry of Education, which facilitates interaction between schools and families. Despite the positive initial reception, a final recruitment phase was implemented through peer-to-peer networking within the school’s parent groups to ensure a robust response rate.

### 2.4. Data Analysis

Responses collected via Google Forms were exported to Microsoft Excel to construct the primary database. Each entry was rigorously reviewed to ensure data integrity, checking for errors, omissions, and adherence to the study’s inclusion criteria.

A coding process followed, converting qualitative information into quantitative data to facilitate statistical analysis. Data processing was performed using SPSS (Statistical Package for the Social Sciences), selected for its efficiency in complex calculations and its ability to generate representative graphical data.

The statistical analysis included:

Descriptive Statistics: Frequency tables and measures of central tendency (mean and mode).

Distribution Analysis: Testing for normality of the responses.

Bivariate Analysis: Cross-tabulation and Cramér’s V coefficient to determine the strength of association and the statistical significance of relationships between variables.

To account for potential confounding factors, such as the interaction between educational attainment and nationality, a multivariate logistic regression analysis was performed. This allowed for the calculation of Adjusted Odds Ratios (aOR), ensuring that the associations observed (e.g., between nationality and caries etiology) remained statistically significant even when controlling for socioeconomic variables. The significance level was set at *p* < 0.05.

Operationalization of Variables To ensure consistency throughout the study, the following key variables were defined:

Perceived Etiology of Caries: Assessed whether parents identify caries as an infectious/preventable disease versus a hereditary/unavoidable condition.

Caries Prevention Knowledge: Evaluated understanding of specific actions (hygiene, diet, fluoride) to prevent decay.

Primary Dentition Value: Measured the level of importance parents attribute to the health of baby teeth compared to permanent teeth.

Preventive Habits: Recorded the frequency of dental visits and the timing of the first dental check-up.

### 2.5. Ethical Considerations

The study was conducted in accordance with the Declaration of Helsinki, and approved by the Ethic Committee Name: CEI Provincial de Huelva, Consejería de Salud y Consumo de la Junta de An-dalucía. Approval Code: SICEIA-2025-000126 Approval Date: 3 March 2025. Participation was voluntary, and written informed consent was obtained from parents or legal guardians after providing detailed information about the study’s objectives and procedures.

## 3. Results

### 3.1. Sociodemographic Characteristics of the Participants

The study included a total of 129 parents and guardians. As shown in Table 1, the majority of respondents were born in Spain (69%), while 29.5% were foreign-born. The age distribution of the guardians was concentrated in the 30–49-year range (67.4%). Regarding educational attainment, 51.2% of the sample reported a medium level of education, followed by 29.5% with a low level and 15.5% with a high level.

### 3.2. Influence of Nationality on Dental Attendance and Oral Health Knowledge

Nationality emerged as a significant determinant of both dental attendance and the perception of caries etiology. Spanish-born guardians reported a significantly higher rate of dental visits for their children (97.7%) compared to foreign-born guardians (84.2%), a difference that was statistically significant (*p* = 0.030; Table 2).

Regarding the knowledge of caries prevention and its causes, the results indicated a profound cultural gap. A vast majority of Spanish-born respondents (98.4%) correctly identified behavioral factors—such as deficient brushing and sugar intake—as the primary causes of caries. Only 1 (1.6%) said it was hereditary. In contrast, 71.1% of foreign-born guardians attributed the condition to “infections, diseases, or heredity” (*p* < 0.001; Table 3). This finding supports the hypothesis that nationality is associated with a fatalistic view of oral health.

### 3.3. Educational Attainment and Clinical Priorities

The level of education significantly influenced how guardians perceive the importance of different dental sectors. As detailed in Table 4, there was a statistically significant association between educational attainment and the perceived etiology of caries (*p* = 0.048), with the high-education group showing 100% accuracy in identifying behavioral causes.

Furthermore, educational level was a decisive factor in the focus of oral health concern. Guardians with low (73.7%) and medium (68.2%) education levels showed a marked preference for the anterior sector (aesthetics). Conversely, 75% of guardians with a high level of education prioritized the posterior sector (function), indicating a highly significant association between academic background and functional awareness (*p* < 0.001; Table 5).

Finally, the analysis revealed a specific knowledge gap regarding the complications of neglecting primary teeth. The medium-education group demonstrated the lowest awareness (34.8%), while the high-education group showed a more balanced understanding. This relationship was statistically significant (*p* = 0.047; Table 6), suggesting that information campaigns should specifically target these gaps in middle-income and immigrant sectors.

As a final resume, significant associations were observed between nationality and both dental attendance and perceptions of caries etiology. Spanish-born guardians reported higher dental attendance among children compared to foreign-born guardians (97.7% vs. 84.2%; *p* = 0.030). Additionally, foreign-born guardians were more likely to attribute caries to hereditary or infectious causes, whereas Spanish-born guardians predominantly identified behavioral factors (*p* < 0.001).

Educational attainment was also significantly associated with oral health knowledge. Participants with higher educational levels more frequently identified behavioral causes of caries (100% vs. 86.8% in the low education group; *p* = 0.048). Furthermore, a non-linear relationship was observed for knowledge of complications, with the medium education group demonstrating the lowest awareness of risks associated with primary teeth (34.8%; *p* = 0.047) (Table 7).

## 4. Discussion

Oral health is a broad and multifaceted concept. It encompasses not only the clinical absence of disease but also the understanding of its etiologies, the consequences of neglect, and the fundamental role of periodic professional evaluations. Despite the current reach of oral health promotion, the focus must remain steadfast on prevention, a priority that must be effectively communicated to patients, particularly to the legal guardians of pediatric patients [19,20]. A lack of knowledge or motivation negatively impacts the long-term prognosis of teeth that could otherwise be preserved to fulfill their physiological, aesthetic, and psychological functions in children [21].

In the present study, factors such as nationality, educational attainment, and age of the legal guardian were observed to potentially compromise the oral health expectations of pediatric patients. Our findings align with previous literature suggesting that nationality remains a barrier to oral healthcare. In this study, a significantly higher percentage of foreign-born guardians (15.8%) reported that their children had never visited a dentist, compared to only 2.3% of Spanish-born guardians. Furthermore, only 25.2% of this group prioritize caries prevention through the combination of efficient brushing, dental visits, and flossing. In this context some studies also reflect that migrant people show poor oral health indicators, and this translates into social vulnerability [22].

It is important to note that while nationality and educational level are both significant predictors, our analysis suggests they act as independent determinants. Even after adjusting for educational attainment, foreign-born status remained a significant factor in the persistence of the ‘hereditary caries’ myth. This indicates that the barrier is not merely formal education, but rather a cultural and linguistic gap in how health information is disseminated and internalized in different communities.

Furthermore, a significant portion of the foreign-born group (71.1%) believes that caries is caused by infections, systemic diseases, or heredity. In contrast, among Spanish-born respondents, only 1.6% said it was hereditary. A stark difference emerged regarding caries etiology knowledge: 73.0% of Spanish-born respondents correctly identified efficient brushing, dental visits, and flossing as primary preventive measures. Moreover, only 1.6% of this group attributed caries to heredity or infections, while 68.5% correctly identified deficient brushing, poor diet, and excessive sugar intake as the primary causes. In the same way other authors have shown that many demographic and socioeconomic variables are strong determinants of parental ratings of child oral health [23].

These disparate percentages compel us to ask: what is the root of this gap? Do religious beliefs, ethnicity, or cultural backgrounds influence oral health perceptions? While oral health campaigns are frequently conducted in schools, they must be reinforced at home [24]. However, if the population remains unaware of the long-term sequelae of poor oral health in childhood, preventive measures will continue to be undervalued. Indeed, 44.1% of all respondents admitted to being unaware of the consequences of neglecting their children’s dental care (18.9% foreign-born and 25.2% Spanish-born).

The influence of parental age was also analyzed, considering the massive influx of health information via social media. Notably, 97.7% of respondents under the age of 50 recognized the importance of brushing primary teeth, whereas only 0.8% of those over 50 dismissed its importance. Nevertheless, no significant correlation was found between age and the motivation to care for their children’s teeth. The majority (72.9%) cited “general health” as their primary motivator, while only 3.1% focused solely on “appearance.” Not every study done reached the same conclusion [25].

These findings suggest that parental age is not a decisive factor in oral health care; rather, there is a general, positive trend toward valuing oral health as an integral part of overall well-being. However, as discussed by Humeres-Flores et al. [19], while guardians value primary teeth, they often place a higher priority on permanent dentition. Our study echoes this sentiment: only 40.3% deemed it necessary to invest in treatments for primary teeth, while 43.4% would only do so if a specific problem arose.

Our findings in Palos de la Frontera align with international research:

Simpson de Paula et al. (Brazil, 2022): Highlighted that higher parental educational attainment correlates with a lower prevalence of caries in children [26]. Vázquez et al. (Chile, 2015): Emphasized the role of teachers in prevention and urged guardians to strengthen the “family-school-oral hygiene” bond through educational workshops [18]. While Vargas Castañeda et al. (Peru, 2022) concluded that sociodemographic and cultural characteristics directly impact preschool oral health, suggesting that successful prevention requires changing parental attitudes toward the value of primary dentition [27].

Ultimately, these results raise critical questions for global public health: Our findings demonstrate that the disparity in oral health knowledge is specifically linked to nationality and education level. Rather than a global lack of information, the results suggest a failure in the local adaptation of preventive messages for migrant and medium-education groups.

To improve the epidemiological landscape of pediatric oral health, a global consensus may be necessary to overcome cultural and racial barriers. Schools must integrate oral health into their “healthy lifestyle” frameworks. In an era of high information accessibility, pediatric patients would benefit significantly from guardians who possess both the knowledge and the motivation required to ensure optimal health for their offspring.

Based on the bivariate analysis of the master tables, it can be concluded that the guardian’s nationality and educational attainment are the primary determinants of oral health literacy in the studied population. A profound cultural gap exists where foreign-born status is significantly associated with the persistence of the “hereditary caries” myth (*p* < 0.001) and a lower dental attendance rate (*p* = 0.030).

Conversely, educational level acts as a catalyst for functional awareness: while guardians with low or medium education levels prioritize the aesthetics of the anterior sector (*p* < 0.001), those with higher education demonstrate a mature understanding of oral health by prioritizing the function and health of the posterior sector (75.0%), while also showing a unanimous recognition that caries is a behavior-dependent disease (*p* = 0.048).

Particularly relevant is the “valley effect” detected in guardians with a medium educational level, who exhibited the highest lack of knowledge regarding complications in primary teeth (65.2%, *p* = 0.047). This finding suggests that public health programs must transcend the “high vs. low education” dichotomy and design communication strategies that specifically target information gaps in middle-income sectors and immigrant populations as it has been mentioned before in other similar studies [28,29].

Ultimately, the transition from a fatalistic (heredity) and aesthetic view toward a preventive and functional perspective is a process mediated by education, justifying the implementation of personalized educational interventions in school and primary care settings to improve children’s oral health.

This study has research implications because our results highlight a significant ‘knowledge gap’ in parents with medium educational levels and migrant backgrounds regarding primary dentition [28]. Future research should employ qualitative methodologies, such as focus groups, to explore the underlying cultural beliefs that sustain the myth of hereditary caries. Additionally, longitudinal studies are needed to evaluate if improving parental literacy through targeted interventions directly correlates with a reduction in the caries index of their children.

On the other side, there are practical implications. For clinical pediatric dentistry, these findings emphasize the need for a more culturally competent communication. Clinicians should not assume that parents with basic or medium education understand the systemic consequences of neglecting primary teeth. Practical tools, such as visual aids and simplified instructional materials in multiple languages, should be integrated into routine visits to overcome the identified linguistic and cultural barriers.

To finish we have also seen in our research some policy implications. From a public health perspective, policies must move beyond universal school-based campaigns toward more personalized, community-level interventions. Health authorities should integrate oral health educators into social services and adult education centers (such as the one studied here) to reach migrant populations. Furthermore, public health messaging should prioritize the ‘functional and preventive’ value of the posterior dentition over aesthetic concerns to realign parental priorities with clinical needs [28,29,30].

Several limitations must be acknowledged. First, the use of non-probabilistic convenience sampling in a single educational center limits the generalizability (external validity) of the results to the entire population of Palos de la Frontera or other vulnerable contexts in Andalusia. Second, the reliance on self-reported data from a questionnaire may introduce social desirability bias, where parents might overreport positive hygiene habits or dental attendance.

Additionally, while the response rate was high (over 65%), those who chose not to participate might have different levels of oral health literacy, potentially leading to a self-selection bias. Finally, the cross-sectional design allows us to identify associations between nationality, education, and knowledge, but it does not establish causal relationships. Despite these restrictions, the study provides valuable and specific insights into a demographic group that is often underrepresented in national health surveys.

## 5. Conclusions

Factors such as nationality and educational attainment significantly influence parental knowledge of early childhood oral health. Respondents born outside Spain and those with lower education levels exhibit a marked lack of information, frequently attributing caries to heredity rather than behavioral factors like diet and hygiene. This highlights a critical socioeconomic gap in access to preventive dental education.

While most parents recognize the importance of oral hygiene for general health, a concerning tendency remains to undervalue primary dentition compared to permanent teeth. Since many guardians only seek treatment when a clinical problem arises, it is essential to strengthen “family-school” preventive programs to overcome cultural barriers and ensure early dental care becomes a universal priority.

## Figures and Tables

**Table 1:** Sample distribution by nationality, parent’s age, child’s age and guardian’s educational attainment.

Variable	Guardian’s Nationality			Guardian’s Age (Years)			Child’s Age (Years)			Guardian’s Educational Attainment				Total Sample
	Spanish	Foreign	Missing Data	<29	30–49	>50	3 y	4 y	5 y	Low	Medium	High	Missing Data	
Frequency (Fr)	89	38	2	41	87	1	43	46	40	38	66	20	5	129
Percentage (%)	69.0	29.5	1.6	31.8	67.4	0.8	33.3	35.7	31.0	29.5	51.2	15.5	3.9	100.0

**Table 2:** Distribution and relation between parent’s nationality and visit to dentist. * *p* < 0.05 means statistical significance.

Variable: Has the Child Ever Visited a Dentist?	Spanish-Born (*n* = 87)	Foreign-Born (*n* = 38)	*p*-Value
Yes [*n* (%)]	85 (97.7%)	32 (84.2%)	0.030 *
No [*n* (%)]	2 (2.3%)	6 (15.8%)	

**Table 3:** Relationship between nationality and caries etiology knowledge. * *p* < 0.05 means statistical significance.

Variable	Spanish (*n* = 89)	Foreign (*n* = 38)	*p*-Value
Prevents caries with brushing/flossing/visits	65 (73.0%)	10 (26.3%)	0.013 *
Perceives caries as behavioral (Sugar/Hygiene)	61 (68.5%)	11 (28.9%)	0.014 *
Perceives caries as hereditary/infections	1 (1.6%)	27 (71.1%)	<0.001 *

**Table 4:** Relationship between educational level of guardians and caries prevention. * *p* < 0.05 means statistical significance.

Educational Level	Infections, Diseases, and/or Heredity (*n*)	Poor Brushing, Diet, and Excess Sugar (*n*)	Total (*n*)	*p*-Value
Low	5 (13.2%)	33 (86.8%)	38	0.048 *
Medium	2 (3.0%)	64 (97.0%)	66	
High	0 (0.0%)	20 (100.0%)	20	
Total	7	117	124	

**Table 5:** Relationship between educational level and aesthetics concerns, * *p* < 0.05 means statistical significance.

Educational Level	Anterior Sector (Aesthetics)	Posterior Sector (Function)	Total (*n*)	*p*-Value
Low	28 (73.7%)	10 (26.3%)	38	<0.001 *
Medium	45 (68.2%)	21 (31.8%)	66	
High	5 (25.0%)	15 (75.0%)	20	
Total	78	46	124	

**Table 6:** Relationship between educational level and knowledge of complications when not treating primary teeth. * *p* < 0.05 means statistical significance.

Variable: Awareness of Complications from Neglecting Primary Teeth	Low Education (*n* = 38)	Medium Education (*n* = 66)	High Education (*n* = 20)	*p*-Value
Correct Knowledge [*n* (%)]	16 (42.1%)	23 (34.8%)	9 (45.0%)	0.047 *
Incorrect/Lack of Knowledge [*n* (%)]	22 (57.9%)	43 (65.2%)	11 (55.0%)	

**Table 7:** Relational matrix: Impact of nationality and education on oral health knowledge. * *p* < 0.05 means statistical significance.

Independent Variable	Key Outcome (Dependent Variable)	Primary Finding	*p*-Value
Nationality	Dental Attendance	Spanish-born guardians show significantly higher access (97.7%) vs. foreign-born (84.2%).	0.030 *
Nationality	Caries Etiology	71.1% of foreign-born guardians attribute caries to heredity/infections, while 98.4% of Spanish-born attribute it to behavior.	<0.001 *
Educational Attainment	Caries Etiology	100% of the High Education group correctly identifies behavioral causes vs. 86.8% in the Low Education group.	0.048 *
Educational Attainment	Knowledge of Complications	A non-linear “gap” exists: Medium Education group has the lowest knowledge (34.8%) of primary tooth risks.	0.047 *

## Data Availability

Due to the dataset contains material subject to internal evaluation and confidentiality considerations, access to the data can be granted by the corresponding authors upon reasonable request and after ensuring compliance with the applicable ethical and institutional requirements.
